# Supplementary material for: Genetic Structure of the Ca Rater Mallorquí Dog Breed Inferred by Microsatellite Markers
Source: Animals (Basel). 2022 Oct 11;12(20):2733. doi: 10.3390/ani12202733 (PMC9597762; doi:10.3390/ani12202733)
Supplement: Supplementary file 1 [file animals-12-02733-s001.zip › animals-1911942-supplementary.pdf]

Table S1. Microsatellites used, chromosome, fluorochrome, 5′ and 3′ sequence of the primers, size range in pb and multiplex reaction.

Name	Chromosome	Fluorochrome	5'–3' Forward	5'–Reverse	Size range (pb)	Multiplex
CXX279	22	HEX	(30A) TGCTCAATGAAATAAGCCAGG	GGCGACCTTCATTCTCTGAC	120–135	P1
FH2054	12	6-FAM	GCCTTATTCATTGCAGTTAGGG	ATGCTGAGTTTTGAACTTTCCC	140–170	P1
FH2848	2	HEX	TTTTTTTTTTTTTTTCAAAACCAACCCATTCA CTC	GTCACAAGGACTTTTCTCCTG	240–270	P3
AHT121	13	6-FAM	TATTGCGAATGTCACTGCTT	ATAGATACACTCTCTCTCCG	80–110	P1
AHT130	36	NED	GTTTCTCTCCCTTCGGGTTC	GACGTGTGTTCACGCCAG	110–140	P3
AHT137	11	HEX	TACAGAGCTCTTAACTGGGTCC	CCTTGCAAAGTGTCATTGCT	120–150	P3
AHT171	6	NED	AGGTGCAGAGCACTCACTCA	CCCATCCACAGTTCAGCTTT	220–245	P2
AHTh260	16	HEX	CGCTATACCCACACCAGGAC	CCACAGAGGAAGGGATGC	240–255	P2
AHTk211	26	HEX	TTAGCAGCCGAGAAATACGC	ATTCGCCCGACTTTGGCA	90–105	P1
AHTk253	23	6-FAM	ACATTTGTGGGCATTGGGGCTG	TGCACATGGAGGACAAGCACGC	280–290	P1
INRA121	21	NED	ATGTAGTTGAGATTTCTCCTACGG	TAATGGCTGATTTATTTGGTGG	85–110	P1
INU005	33	6-FAM	CTTTCTACCAGCAAGGTTAC	TTCCCATTTAATTGCCTCT	105–140	P5
INU030	12	6-FAM	GGCTCCATGCTCAAGTCTGT	CATTGAAAGGGAATGCTGGT	145–155	P4
INU055	10	HEX	CCAGGCGTCCCTATCCATCT	GCACCACTTTGGGCTCCTTC	205–230	P4
REN105L03	11	HEX	GGAATCAAAAGCTGGCTCTCT	GAGATTGCTGCCCTTTTTACC	230–245	P3
REN162C04	7	HEX	TTCCCTTTGCTTTAGTAGGTTTTG	TGGCTGTATTCTTTGGCACA	175–220	P2
REN169018	29	NED	CACCCAACCTGTCTGTTCCT	ACTGTGTGAGCCAATCCCTT	145–180	P3
REN169D01	14	6-FAM	AGTGGGTTGCAAGTGGAAC	AATAGCACATCTTCCCCACG	185–220	P3
REN247M23	15	HEX	TGGTAACACCAAGGCTTTCC	TGTCTTTTCCATGGTGGTGA	265-285	P3
REN54P11	18	6-FAM	GGGGGAATTAACAAAGCCTGAG	TGCAAATTCTGAGCCCCACTG	220–250	P2
REN64E19	34	HEX	(30A) TGTATTTTAATGTGGCAGTTT	GACAAGGACAGGCAATACAGT	160–200	P3
0123rd	1	PET	CACGGACGCAACACGATTTA	CTCCTGACGCAGCAGTTGTC	175–220	P6
0176rd	2	FAM	TGGCTTGGCAACATTGTCTC	ACCTGGGATTCTCTCGGTCA	335–400	P7
0323rd	3	NED	GGAAGCAGCTGGGTTCCTAA	GTTTTCCATGCCCAACTATTTTTGAA	300–320	P7
0669rd	6	NED	TTGCCGAGATCACTCAAGGA	AATTCTGTGCCCCAAAGTGG	340–350	P7
0914rd	9	FAM	TGCATGGTCACAACATCAG	GCACACAAAATTGTGCGGATA	280–295	P7
0959rd	10	NED	CCAGCCAGATGCAAACATTG	GCTCATGTGGTGTTTTTGATG	260–280	P6
1055rd	11	VIC	CCCAAGCTGGGAAGACAAAA	GGGTGGATTTAGGGTGGACA	215–235	P6
1257rd	13	VIC	TCACCTTCTGGATGGGAACC	ATCCTGCAGTTGCTGTGCTG	245–270	P6
1404rd	15	FAM	AGGGCTGTTTGGAGGAACAA	GTTTCTTTGGTCTGACATGAGGGGAC A	135–160	P6
1878rd	21	FAM	TGCCATAAATGCCCAGAACA	TGCCACCTGGCAGTCTTATG	245–270	P6
2469rd	31	FAM	GTGCACTTTGCAAACCCTGA	TTGTAAGCAGGGGCAAGTGA	310–330	P7
2642rd	35	FAM	GTTCCATGCATGCTGACACA	GGGGTGAGAATGATGGTGGT	85–110	P6

Table S2. Genetic diversity parameters in Ca Rater Mallorquí. Microsatellite, number of alleles detected (NA), effective number of alleles (Ae), observed heterozygosity (Ho), expected heterozygosity (He), polymorphic information content (PIC), FIS and its confidence interval, and HWE deviations after Bonferroni correction.

Microsatélite	NA	Ae	HO	HE	PIC	FIS	FIS (IC)	HW
AHT121	9	4,53	0,813	0,785	0,756	-0,349	(-0,138-0,064)	NS
AHT137	7	3,86	0,698	0,747	0,710	0,066	(-0,909-0,204)	NS
AHTh130	10	5,98	0,783	0,840	0,812	0,068	(-0,064-0,177)	NS
AHTh171	9	5,21	0,828	0,815	0,782	-0,017	(-0,121-0,080)	NS
AHTh260	7	2,68	0,688	0,631	0,573	-0,090	(-0,231-0,045)	NS
AHTK211 1	5	3,59	0,683	0,727	0,675	0,062	(-0,076-0,196)	NS
AHTK253	5	3,82	0,778	0,744	0,694	-0,046	(-0,177-0,080)	NS
CXX279	6	5,64	0,758	0,829	0,797	0,086	(-0,033-0,209)	*
FH2054	8	3,89	0,719	0,749	0,705	0,041	(-0,103-0,176)	NS
FH2848	7	3,68	0,661	0,734	0,687	0,099	(-0,055-0,234)	NS
INRA21	7	3,06	0,688	0,679	0,623	-0,013	(-0,172-0,142)	NS
INU005	5	2,42	0,656	0,591	0,532	-0,111	(-0,285-0,054)	NS
INU030	4	2,52	0,500	0,607	0,553	0,178	(0,012-0,313)	NS
INU055	6	2,01	0,531	0,507	0,461	-0,049	(-0,209-0,120)	NS
REN105L03	9	3,46	0,703	0,717	0,674	0,019	(-0,139-0,165)	NS
REN162C04	6	4,49	0,844	0,783	0,741	-0,078	(-0,192-0,036)	NS
REN169D01	8	5,60	0,688	0,828	0,799	0,171	(0,036-0,292)	NS
REN169O18	9	5,34	0,844	0,819	0,790	-0,030	(-0,137-0,066)	NS
REN247M23	6	3,17	0,656	0,690	0,638	0,049	(-0,095-0,280)	NS
REN54P11	11	2,73	0,563	0,639	0,618	0,120	(-0,018- 0,244)	NS
REN64E19	8	2,93	0,597	0,664	0,629	0,102	(-0,069-0,261)	NS
0123RD	6	3,22	0,656	0,695	0,654	0,057	(-0,075- 0,183)	NS
0176RD	8	4,00	0,780	0,757	0,714	-0,031	(-0,1461-0,067)	NS
0323RD	4	2,39	0,645	0,587	0,526	-0,100	(-0,282- 0,056)	NS
0669RD	11	7,07	0,964	0,866	0,843	-0,113	(-0,188- (-0,042))	*
0914RD	4	1,94	0,353	0,489	0,453	0,280	(0,022-0,510)	*
0959RD	2	1,62	0,161	0,386	0,310	0,584	(0,324-0,803)	***
1055RD	2	1,34	0,234	0,255	0,221	0,081	(-0,156-0,365)	NS
1257RD	6	2,93	0,581	0,664	0,601	0,127	(-0,031-0,279)	NS
1404RD	7	3,38	0,594	0,710	0,668	0,165	(-0,008-0,325)	NS
1878RD	6	3,60	0,581	0,728	0,681	0,204	(0,044-0,363)	NS
2469RD	4	2,93	0,661	0,665	0,591	0,005	(-0,167-0,152)	NS
2642rd	6	2,98	0,734	0,669	0,611	-0,098	(-0,249-0,039)	NS
Average	***6,61***	***3,58***	***0,655***	***0,685***	***0,640***	***0,044***	***(-0,0002-0,073)***	

NS: Non-significant. *: P value <0,05, **: P value <0,01, ***: P value < 0,001

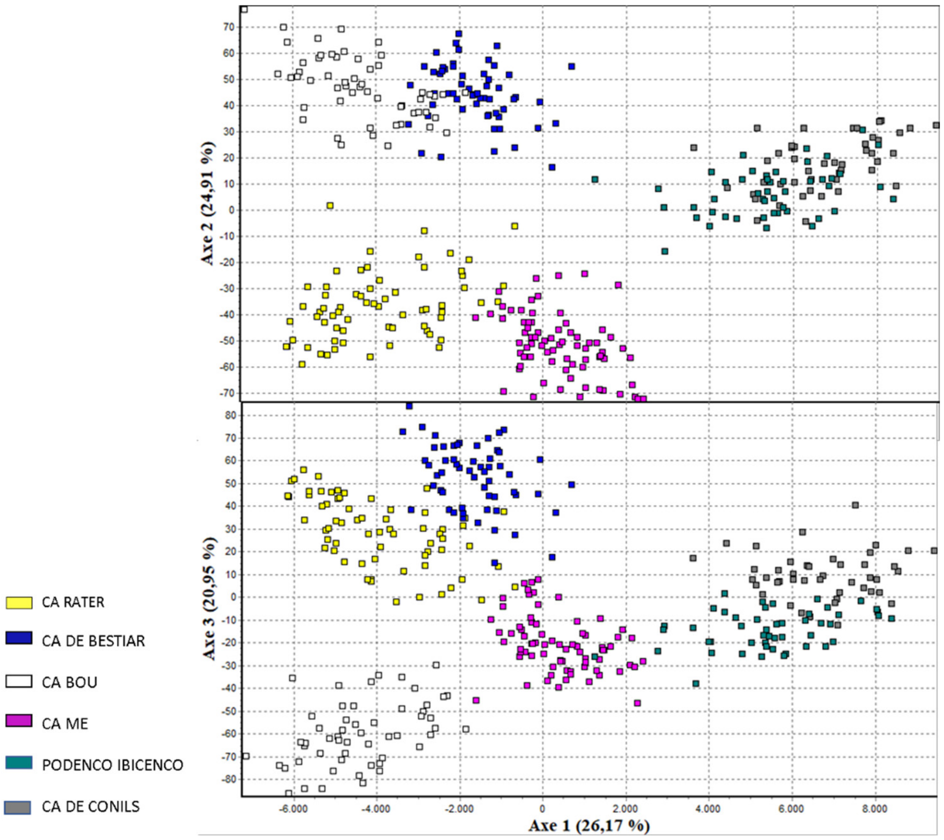

Figure S1. Spatial representation of genetic distances among the breeds analysed, from the first three axes obtained in the factorial analyses of correspondence based on microsatellite data. Values between brackets on axes represent the contribution in % of each axis to total inertia.

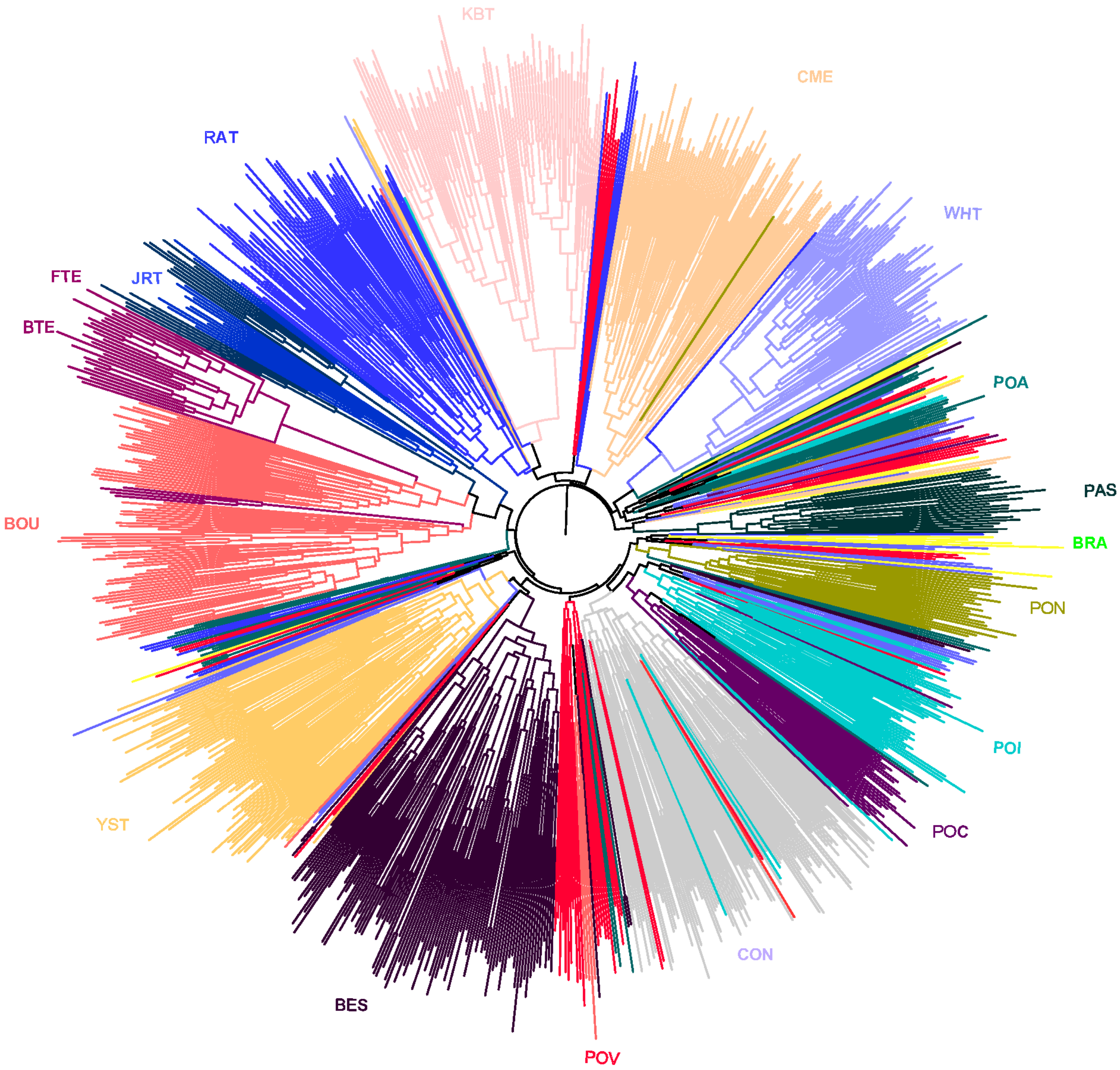

Figure S2. Individual Neighbour-joining representation of D_{AS} genetic distances between 18 dog breeds based on microsatellite data. Breed acronyms are as defined in Table 1.
